# Comparing the Consumption of CPU Hours with Scientific Output for the Extreme Science and Engineering Discovery Environment (XSEDE)

**DOI:** 10.1371/journal.pone.0157628

**Published:** 2016-06-16

**Authors:** Richard Knepper, Katy Börner

**Affiliations:** 1 School of Informatics and Computing, Indiana University, Bloomington, Indiana, United States of America; 2 Department of Information and Library Science, School of Informatics and Computing, Indiana University, Bloomington, Indiana, United States of America; 3 Indiana Network Science Institute, Indiana University, Bloomington, Indiana, United States of America; National Research Council of Italy (CNR), ITALY

## Abstract

This paper presents the results of a study that compares resource usage with publication output using data about the consumption of CPU cycles from the Extreme Science and Engineering Discovery Environment (XSEDE) and resulting scientific publications for 2,691 institutions/teams. Specifically, the datasets comprise a total of 5,374,032,696 central processing unit (CPU) hours run in XSEDE during July 1, 2011 to August 18, 2015 and 2,882 publications that cite the XSEDE resource. Three types of studies were conducted: a geospatial analysis of XSEDE providers and consumers, co-authorship network analysis of XSEDE publications, and bi-modal network analysis of how XSEDE resources are used by different research fields. Resulting visualizations show that a diverse set of consumers make use of XSEDE resources, that users of XSEDE publish together frequently, and that the users of XSEDE with the highest resource usage tend to be “traditional” high-performance computing (HPC) community members from astronomy, atmospheric science, physics, chemistry, and biology.

## 1. Introduction

The XSEDE project (http://www.xsede.org) is the most advanced large-scale computational infrastructure in that U.S. serving basic science research. The charge of the National Science Foundation (NSF) to XSEDE is to support the computational needs of basic science by integrating advanced digital resources and services. Conforming with this mission, XSEDE’s infrastructure supports the computational and data needs of science in a broad range of fields, providing a set of tools that accelerate the pace of scientific research. XSEDE is used both by scientists at the limits of their field who need advanced computational capacity and those who are in need of resources beyond what their local institutions can provide. Access to XSEDE is granted by a peer-reviewed allocation system for computer time, data storage, and consulting services.

The collection of resources and services currently provided by the XSEDE framework saw its genesis in the NSF Supercomputer Centers program started in 1985, and continued its evolution through the National Partnership for Advanced Computational Infrastructure (NPACI), and the TeraGrid programs.

Each new program brought major advancements in the design and usage of cyberinfrastructure in support of research. The Centers program established a number of supercomputing centers which would lead the development of large systems. From the mid- 1980s through the 1990s, the PACI and NPACI programs began the work of connecting these centers in a meaningful way. Starting in 2001, the TeraGrid developed a central organizational structure for obtaining accounts and allocations on systems, and worked to establish common user environments, including a standard software set, as well as training and education programs [1].

Replacing the TeraGrid in 2011, the XSEDE project integrates and broadens the reach of systems by providing services which bridge beyond the project and its usual members to new institutions and new fields of science.

**Fig 1:**
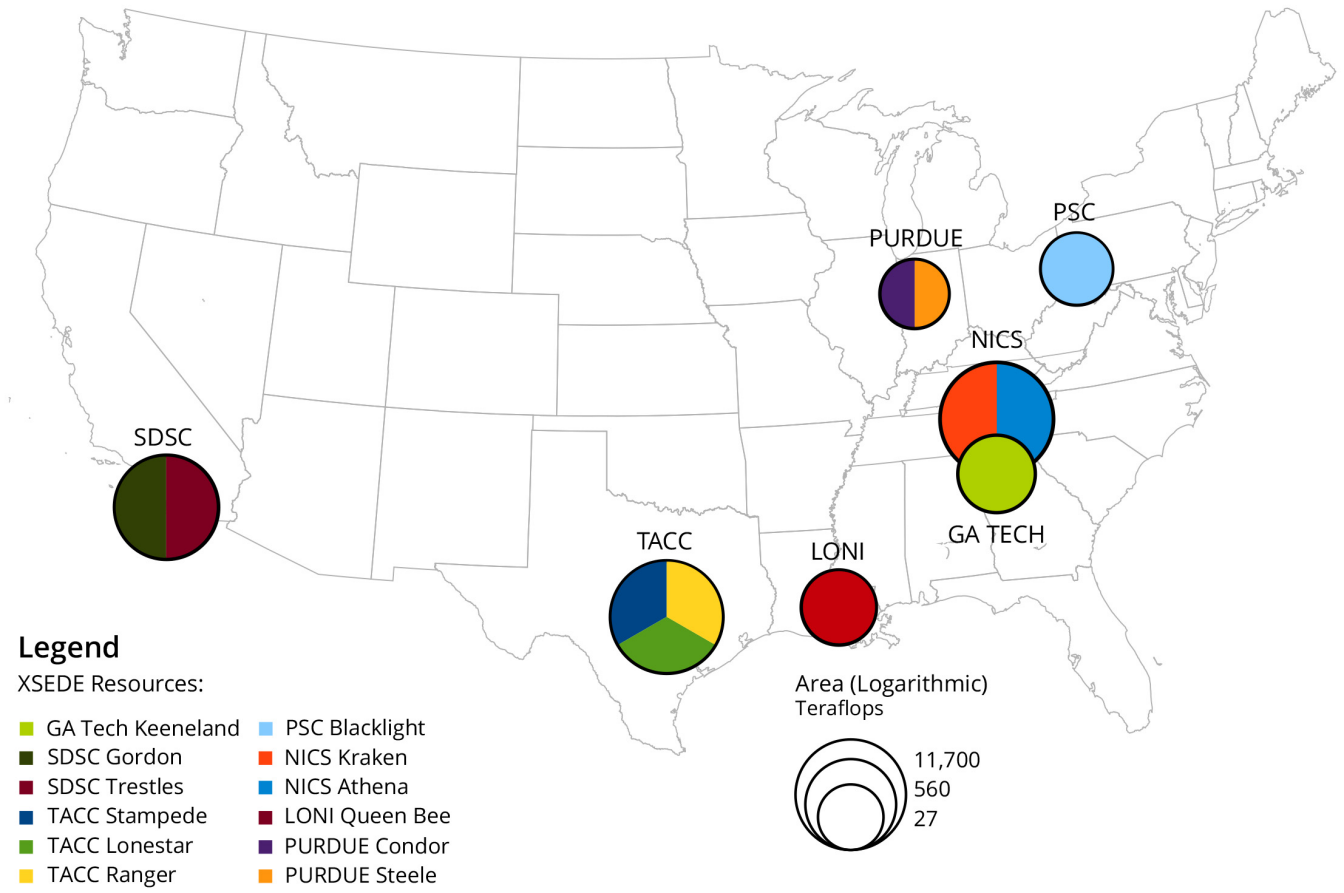
Service Provider Resources, sized by Teraflops.

XSEDE’s approach is to provide a framework which is not tied to any one particular supercomputer or supercomputing center, but which provides a common set of services that allow authentication, resource allocation, training, documentation, and application support. Resources are provided by *Service Providers* (XSEDE terminology) who frequently are funded to implement individual systems that can be made accessible to the XSEDE framework. The *Service Providers* may come and go out independent of the overall XSEDE project timeline and may have varying levels of interoperability with XSEDE. This arrangement allows XSEDE to be more flexible within the funding structures for supercomputing resources, which frequently do not align with project timelines overall. However, this flexibility also creates a higher level of complexity within the XSEDE project. Not all of the partner institutions provide supercomputer resources, some provide other XSEDE services, and not all of the *Service Providers* are listed as partner institutions. The XSEDE systems that make up the top-12 *Service Provider* systems that provide the most widely used computational resources (in terms of CPU hours) between 2011 and 2015 [2] are show in Fig 1.

Table 1 lists all XSEDE computational resources by different service providers that were available during the 2011–2015 period [3]. Systems in the table are ranked by peak Teraflops, denoting the overall computing power of each system. Note that the top resource at TACC, i.e., TACC Stampede, provides almost as many cycles as all other resources combined.

**Table 1:** XSEDE Service Provider Resources during 2011–2015.

Site & Name	Peak Teraflops
**TACC Stampede**	9,600
**NICS Kraken**	1,170
**LSU SuperMIC**	925
**TACC Ranger**	504
**SDSC Gordon**	341
**TACC Lonestar**	311
**NICS Darter**	248
**GA Tech Keeneland**	255
**NICS Athena**	166
**NCSA Forge**	153
**SDSC Trestles**	100
**Purdue Steele**	67
**Purdue Condor**	60
**TACC Maverick**	59
**LONI Queen Bee**	50
**NCSA Lincoln**	47
**IU Big Red**	40
**PSC Blacklight**	36
**TACC Longhorn**	20
**NCSA Ember**	16
**NICS Nautilus**	8
**PSC Pople**	5
**SDSC Dash**	5
**NCAR Frost**	5
**SDSC Comet**	2
**TACC Spur**	1
**OSG**	0**

** OSG as a collection of resources used for high-throughput computing rather than a single high-performance system, does not have a peak teraflops measurement.

Each XSEDE Service Provider needs to meet a certain level of service that define the integration with XSEDE accounts and accounting processes, software, plus job scheduling resources. The XSEDE Service Provider Forum, made up of XSEDE management and Service Provider (SP) leadership, establishes the three service tiers as follows [4]:

- Tier 1: SPs are the most highly-integrated with XSEDE, providing a dedicated 10Gb/s connection to the XSEDEnet service, leveraging the XSEDE accounts and allocation system, installing XSEDE client and server software, and providing training and educational opportunities to XSEDE users.
- Tier 2: SPs provide similar functionalities as Tier 1, with the removal of the requirement to provide XSEDEnet connections, training and educational materials, and fewer requirements to install XSEDE software and services.
- Tier 3: SPs are free from nearly all requirements, other than the provision of basic information about the resource to XSEDE to be published in the XSEDE Information Services.

Systems such as the Stampede system at TACC, Kraken at NICS, Comet and Gordon at SDSC, and Blacklight at PSC are Tier 1 systems. The Mason system at IU, Blue Waters at NCSA, SuperMIC at LSU, and Open Science Grid systems are Tier 2 systems. Current Tier 3 systems are MSI at University of Minnesota and Rutgers at Rutgers University.

The rest of the paper is organized as follows: The subsequent section discusses related work on analyzing resource usage data and publication data to understand the impact of cyberinfrastructure resources on progress in research. Next, we detail the XSEDE, TeraGrid, and publication data used in this study as well as data cleaning and processing applied. Then, we introduce the diverse methods applied to render resource usage data and publication data into insights. The results section discusses key findings and their implications. We conclude with a discussion section that also covers planned future work.

## 2. Related Work

A number of resource utilization and input-output studies have been conducted for the TeraGrid and XSEDE projects. Using the TeraGrid database of accounts, allocations and CPU charges, [5] examined resource utilization on the basis of individual workload characteristics, finding that patterns of job submissions to TeraGrid systems do not necessarily correspond to usage modalities, that is, submitters such as gateways that might be expected to submit jobs across a wide range of resources, frequently submit jobs to a single resource rather than taking advantage of the grid as a whole. In contrast to a true grid, in which middleware submit jobs across a wide range of available resources, TeraGrid submissions are largely user-dependent, and they largely reflect the usage policies in place at a particular site. Hart also finds that allocations are successful in controlling the global demand across a number of systems. HPC usage across a federation of systems appears to reflect usage patterns previously displayed on single systems, but the manual balancing of the TeraGrid allocations system creates different patterns of usage on the individual systems. Another study by [6] documents the results of a network analysis of TeraGrid user and project allocations. Results show that large projects frequently make use of multidisciplinary teams and that teams working on TeraGrid allocations are made up of both domain scientists and technical specialists, while smaller groups tend to be populated by domain scientists alone. Computer scientists may be members of a number of projects in varying scientific domains, while domain scientists tend to remain in their area. [7] used information on resource utilization to improve operations by analyzing resource-specific data on throughput together with codes on individual elements of XSEDE, to characterize project activities, and to identify under-performing codes. The authors show that molecular biosciences are rapidly gaining prominence in the TeraGrid and XSEDE environments, and that they represent a significant departure in usage modality (many cycles on a smaller number of cores) as opposed to traditional HPC domains such as astronomy, physics, and atmospheric sciences, in which large analyses are employed that utilize a large number of cores. In order to collect this data, the team created the data service which informs significant amounts of the present study.

[8] propose a number of measures for improving impact assessment for the use of TeraGrid for its scientific users, noting that the current measures (such as number of users, usage data, and publication information) provide information about outputs of the system, but not necessarily scientific outcomes. This team, established as a “Requirements Analysis Team” by TeraGrid leadership in order to ascertain requirements that would extend and improve TeraGrid activities, recommended a number of activities that would capture impact on scientific research and knowledge, including improving the proposal system in order to better capture data such as supporting grant funding, adopting the NSF’s practice of keeping a database of “science nuggets” (short description of scientific work done and the contribution of the TeraGrid to the project), and improving survey practices.

[9] conducted a bibliometric analysis of publications supported by TeraGrid and XSEDE allocations, describing the impact of resource utilization on publication frequency. Results show that while at the individual project level, the use of TeraGrid and XSEDE infrastructure does not show a strong positive correlation with impact metrics (e.g., number of publications, number of citations, h-index, and g-index), when usage is aggregated at the field of science (FOS) level, larger allocations are positively correlated with all four of these impact metrics, leading to the conclusion that resources matter in terms of consumption at the aggregate FOS level.

[10] categorize the inputs and outputs of research work based on citations and publications with a focus on the exchange of information across national boundaries. The authors identify knowledge sources and sinks by geolocation, and find that the coastal United States, England, and parts of Central Europe appear to be knowledge sources, while Asia and South America appeared to largely be knowledge sinks (researchers citing others in their publications but not being cited themselves). This geographic exchange of scientific knowledge shows that flows of information can be mapped in order to identify sources and destinations of scientific information.

This work attempts to extend both the resource utilization and publication production perspectives by examining the consumption of resources and the production of publications at the level of organizations and individual project participants in the way that [9] did, but combining resource analysis with the geographical network analysis perspective in [10], representing Service Providers which provide the resources for consumption and project PIs and their associated publications.

## 3. Data Acquisition and Preparation

Three main types of data are used in this analysis: XSEDE user records and project data, XSEDE Metrics on Demand, and XSEDE publications. The data provided by XSEDE covers a span from the inception of the Teragrid Central Database in 2003 through the transition to XSEDE and up to August of 2015. The data described below has been provided by XSEDE staff who create and present metrics for usage and application data, as well as by those who are engaged in project management and documentation of project results to the NSF.

### XSEDE Project Records

The original dataset on projects and users in TeraGrid was compiled with the assistance of the XSEDE accounts management team. A representative of the accounts team ran SQL queries against the XSEDE Central Database (XDCDB), originally the TeraGrid Central Database (TGCDB), which tracks all XSEDE accounts, allocations, and resource usage. The retrieved data covers all user and project information from the inception of the accounting system in 2003 through August of 2015. It includes information for 17,972 resource allocations, comprising a total of 23,286,865,254 CPU Hours, for 4,845 Principal Investigators. XDCDB is populated by information collected by the Account Management Information Exchange (AMIE) system(http://scv.bu.edu/AMIE). All data was provided in comma-separated value files that can be easily processed programmatically.

The project data includes:

- Allocation short name, or Project ID and allocation identifier
- ID and name of Principal Investigator
- ID and name of PI Organization
- ID, organization, and name of resource used in allocation
- Field of science, consisting of 147 specific fields
- CPU hour usage of the allocation
- Base allocation, the initial allocation in service units (allocations can be extended for long-term projects)

An abbreviated sample of the project description data is given in Table 2.

**Table 2:** Project and allocation data from XDCDB.

Allocation	PI ID	PI Organization	Resource	Field	CPU Usage	SUs Allocated
**TG-MCA93S002**	7	UC Santa Barbara	kraken.nics.teragrid	Elementary Particle Physics	507,831	99,563,180
**TG-MCA93S028**	8	UIUC	stampede.tacc.xsede	Biochemistry and Molecular Structure Function	21,299,283	97,955,353
**TG-PHY130005**	1495	Jefferson Lab	stampede.tacc.xsede	Nuclear Physics	18,201,123	83,706,917
**TG-IBN130001**	17937	U Pittsburgh	grid1.osg.xsede	Behavioral and Neural Sciences	257,735	81,456,774
**TG-MCA07S017**	5148	CMU	kraken.nics.teragrid	Nuclear Physics	38,237,878	75,210,479

### XSEDE Metrics on Demand

The XSEDE Metrics on Demand (XDMoD) site (https://xdmod.ccr.buffalo.edu) was developed at the University at Buffalo and is detailed in [11]. It leverages the XDCDB as well as a number of probes which examine the performance of XSEDE resources, including individual application performance. XDMoD includes information which can be explored by a number of means, including by *PI*, *PI Institution*, *Field of Science*, *and Allocation*, among many others. As part of the allocations process, PI’s agree that XDMoD also provides a number of means for organizing and visualizing data about XSEDE. Data from XDMoD can be exported into tractable data formats such as csv, for programmatic manipulation. XDMoD staff provided support in querying and using the XDMoD database. Reports from the XDMoD database allow the aggregation of usage and allocation on a per-project or per-PI basis.

To create maps of XSEDE resource consumption, institution data was matched with a lookup table of latitudes and longitudes provided by XSEDE. There are a few projects, such as the OpenWorm Project, which are virtual organizations that list no location. Exactly four organizations had a latitude and longitude of 0,0 and they were removed from the dataset.

In order to create a time-series representation of utilization, queries to XDMoD were created for each year since the XSEDE project’s start (July 1-June 30, except for 2014–2015, when data was collected on March 7, 2015). A summary of this data can be seen in Table 3. This data is generated by user interactions with XSEDE resources and accounting takes place directly based on accounts used to authenticate and is tied to the XDCDB information, institutional and PI data is understood to be largely correct. The only instance of incorrect information included in this information would be if a user was using another user’s account (a violation of XSEDE policies) or if incorrect information was entered into XDCDB.

**Table 3:** CPU hours, institutions, and PI's by year.

Year	Total CPU Hours Consumed	Number of Institutions	Number of PI’s
**2011**	1,554,673,368	462	1399
**2012**	1,534,421,510	500	1471
**2013**	1,622,752,650	558	1672
**2014**	1,230,029,427	558	1669
2015*	1,159,999,035	571	1706

* Note: data for 2014–2015 was only collected for July 1, 2014 through March 7, 2015

### XSEDE Publication Data

XSEDE staff provided the contents of the XSEDE publications database, which is a self-reported database of publications supported by XSEDE. Individual users of XSEDE record their publications via the XSEDE user portal, where they can be viewed as online user profiles, but also used by XSEDE in order to measure and demonstrate the scientific output of researchers making use of XSEDE. The publications database as provided contains 2,882 submissions, of which 2,660 are associated with XSEDE projects and 222 are recorded as supporting materials for XSEDE allocations requests, which XSEDE staff assert to not be the result of work done on XSEDE, but rather work preliminary to beginning work on the XSEDE project, and these records were removed as they do not represent utilization of XSEDE resources. XSEDE publications data, because it is self-recorded, tends to be messy, and requires some processing. For the purposes of a co-authorship network analysis, the data was reformatted as a bibtex file and author names were unified. Records that were not able to be parsed from the XSEDE data into bibtex were discarded. In all, 1641 total publications were obtained.

## 4. Methods

All of the above data collection and the following analyses were approved by the Indiana University Institutional Review Board on 5/20/2015 under Protocol #1505700624. In order to create a map of the top ten resource users, data on usage by number of CPU Hours used by PI Institution was retrieved from the XDMoD exported into a spreadsheet file. The top 10 institutions were ranked largest to smallest by amount of usage, and usage breakdowns by resource were obtained from XDMoD. For the top 10 institutions, usage at each resource was converted to a percentage of total use and the percentages used to generate pie charts, which were then superimposed over institutions geolocated on a map of the United States.

Using the XSEDE publication data, a co-authorship network was extracted and author names were unified using the Sci2 Tool described in [12]. The resulting co-authorship network has 2,918 author nodes and 6,983 collaboration links. This network was then analyzed with the MST Pathfinder algorithm in order to detect the backbone structure of the network. Weak component analysis was run to identify the largest fully connected component. This resulting graph was analyzed for modularity in Gephi and color was used to indicate what authors belong to what cluster modules.

Usage data from XSEDE project records was analyzed by field of science in order to understand utilization of resource based on field of science. The allocation and usage information project records were transformed into a bipartite network. One set of nodes in the bipartite network is fields of science, and one set is XSEDE resources, with edges weighted by the amount of usage. The resulting bipartite network was loaded in Sci2, edges above 100M CPU hours usage were extracted, and the resulting network rendered as a bipartite network graph.

In order to analyze the usage and publication data in respect to location, the Sci2 Tool was used to read the bibtex file and extract a two-mode network. The two-mode network hast two types of nodes: resources and organizations. Resource nodes have attributes such as location (lat-lon) and capacity (teraflops). Organization nodes have attributes for location and number of publications (aggregated for all users at an individual organization). Organization location is derived from XSEDE’s table of all organizations that use XSEDE. The edges between resources and organizations are weighted by the size of the allocation in CPU usage. The resulting network was analyzed for centrality and node degree distribution using the Network Analysis Toolkit in the Sci2 Tool. Edges above 25M CPU Hours of utilization were extracted from the network and nodes were geolocated by institutional information, and the resulting network overlaid on a map of the United States.

## 5. Results

**Fig 2:**
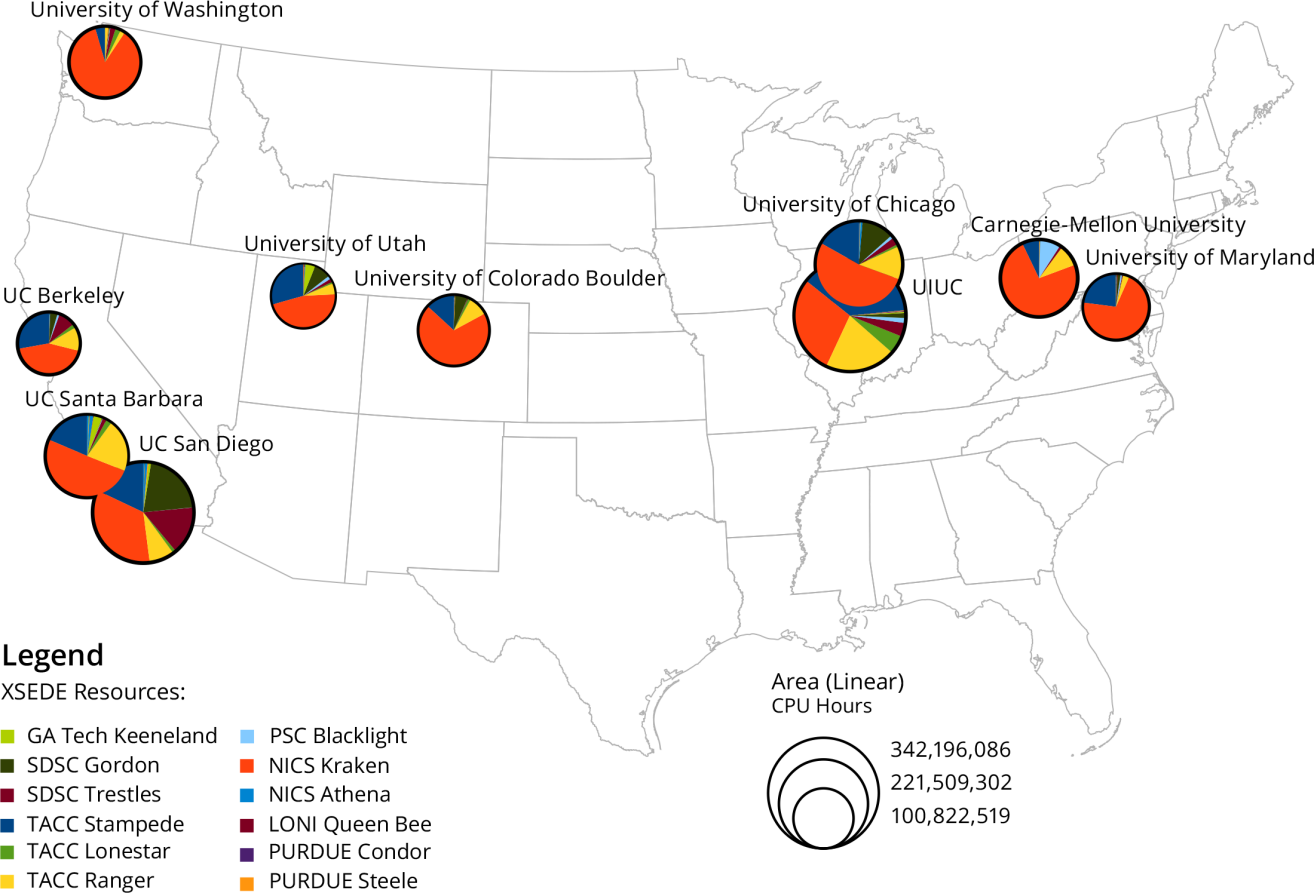
Top 10 Consumers of XSEDE Resources.

Compiling the usage data, it was possible to examine each of the five program years individually and in aggregate. Looking at aggregate usage by PI institution, it appears that a significant amount of CPU hour consumption happens at the institutions which house individual centers in XSEDE: the University of Illinois Urbana-Champaign (UIUC), Carnegie-Mellon University, University of California San Diego—all locations which house Service Providers—first and foremost, with other prominent institutions being University of Chicago, University of California Santa Barbara, University of Washington, and University of Maryland, followed by University of Utah, University of Colorado Boulder, and University of California Berkeley. The top 10 institutions consuming XSEDE resources are shown in Fig 2. In the figure, for each of the pie charts at these institutions, each color represents the proportion of total usage at each of the 12 resources in the legend. For all of these institutions, the NICS Kraken system is the most prominent provider of resources, followed by the Stampede and Ranger systems at TACC. It is also notable that University of California San Diego makes significant usage of local systems, spending roughly 30% of its total CPU hours on the Gordon and Trestles systems, while these resources do not play a significant part in the usage of other sites. It is also apparent that while TACC and NICS provide a significant amount of resources—making up more than half of all CPU hour consumption by the top 10 institutions—there is a limited amount of local usage.

**Fig 3:**
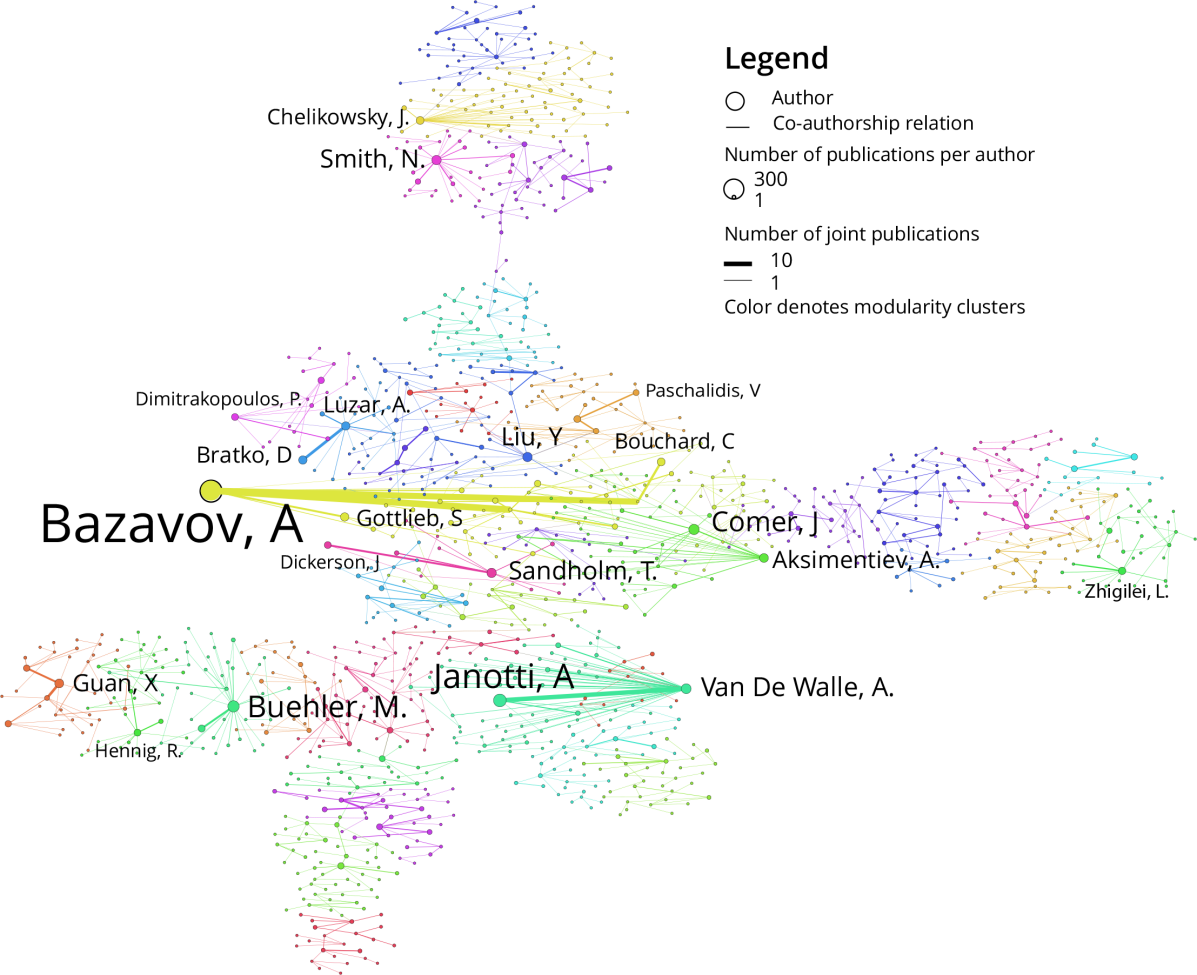
Co-authorship network of XSEDE users.

Fig 3 shows the co-authorship network derived from the 1,641 publications that cite XSEDE resources. As the original network was rather dense, MST Pathfinder network scaling was applied in Sci2 to extract the “backbone” of the strongest co-authorship links. Gephi was used to identify network modularity and to color the nodes by modularity cluster. Nodes in the image are grouped into communities by the number of edges they share within the group versus the expected number of connections for the entire network. Colors in the figure are used to visually distinguish these communities from each other. Edges of the network are weighted by number of coauthored publications, and author nodes are sized by number of publications.

The co-authorship network exhibits considerable interactions between authors—the largest connected subcomponent consists of 1,244 nodes, nearly half of the 2,631 nodes in the network. Average degree for the co-authorship network is 1.998.

**Fig 4:**
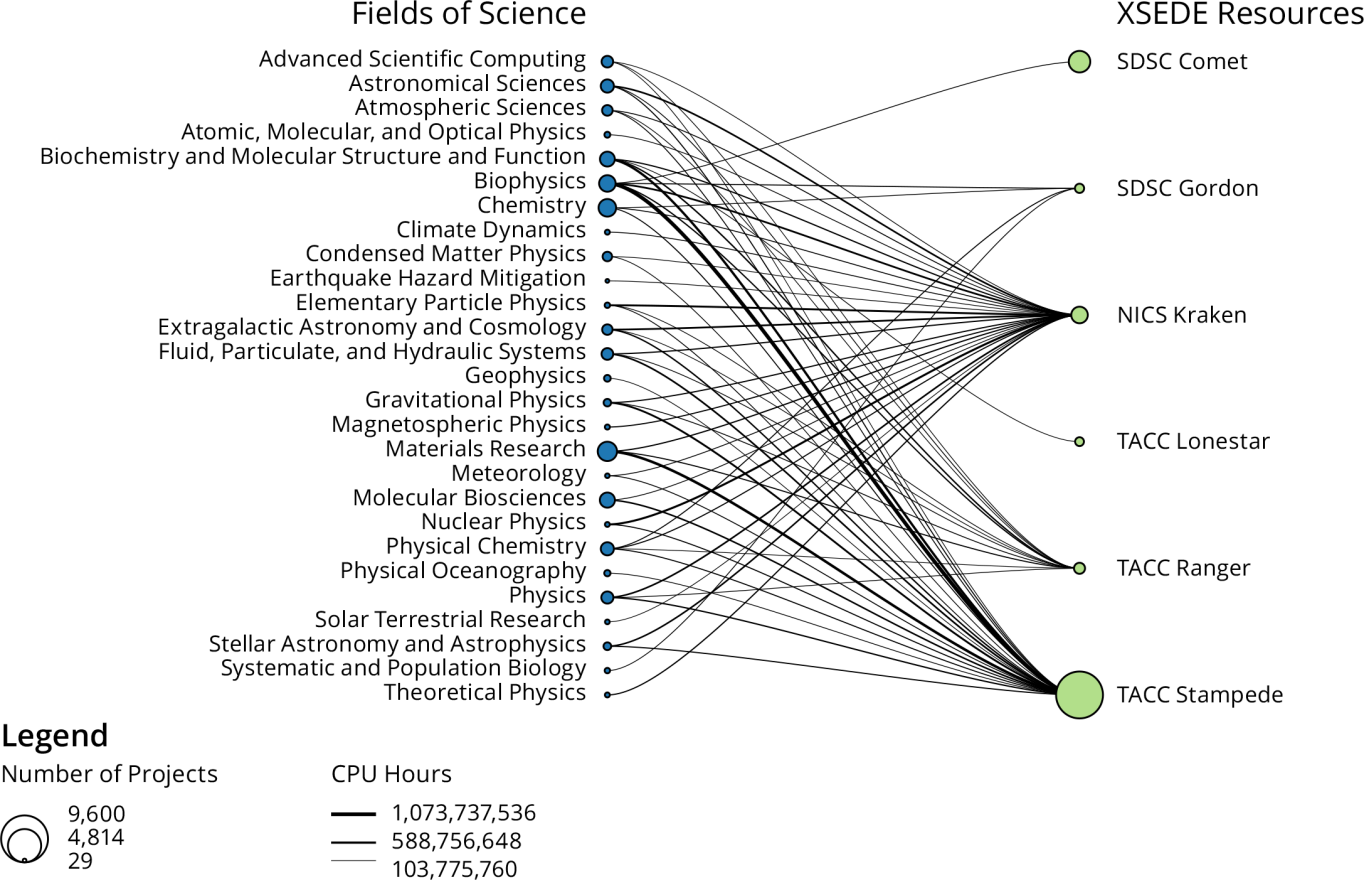
Bipartite field of science and resource network.

The next analysis of XSEDE usage data deals with the top consumers of XSEDE resources grouped by field of science. In the project data there are a total of 150 fields of science making use of 54 resources across the life of the project. Exactly 27 of these 150 fields use more than 100,000,000 CPU hours according to the usage data. These 27 fields were subsequently linked to the XSEDE resources they use. The resulting bipartite network is displayed in Fig 4. Among the top-27 fields of science which make the most use of XSEDE resources are the “traditional” fields such as Physics, Chemistry, Astronomy, Material Research, and Meteorology/Climate Modeling. The figure also shows that some XSEDE resources serve a broad number of fields, while others perform significant amounts of work for a smaller numbers of fields. For example, the NICS Kraken system has links with nearly every one of the fields in Fig 4, while SDSC Comet, SDSC Gordon, and TACC Lonestar have links with only a few fields. Since the cut-off of this network is lower than 100,000,000 CPU Hours, connections in this network represent only the fields of science of the largest projects—a broader range of fields make use of these resources at a lower level of utilization. The “leadership class” systems Stampede and Kraken clearly attract and serve projects from a broad range of fields, while other significant usage for systems such as Comet and Lonestar4 come from single fields of science. The SDSC Comet system is not included in all analyses, as it is a newer system and has had a limited amount of use.

**Fig 5:**
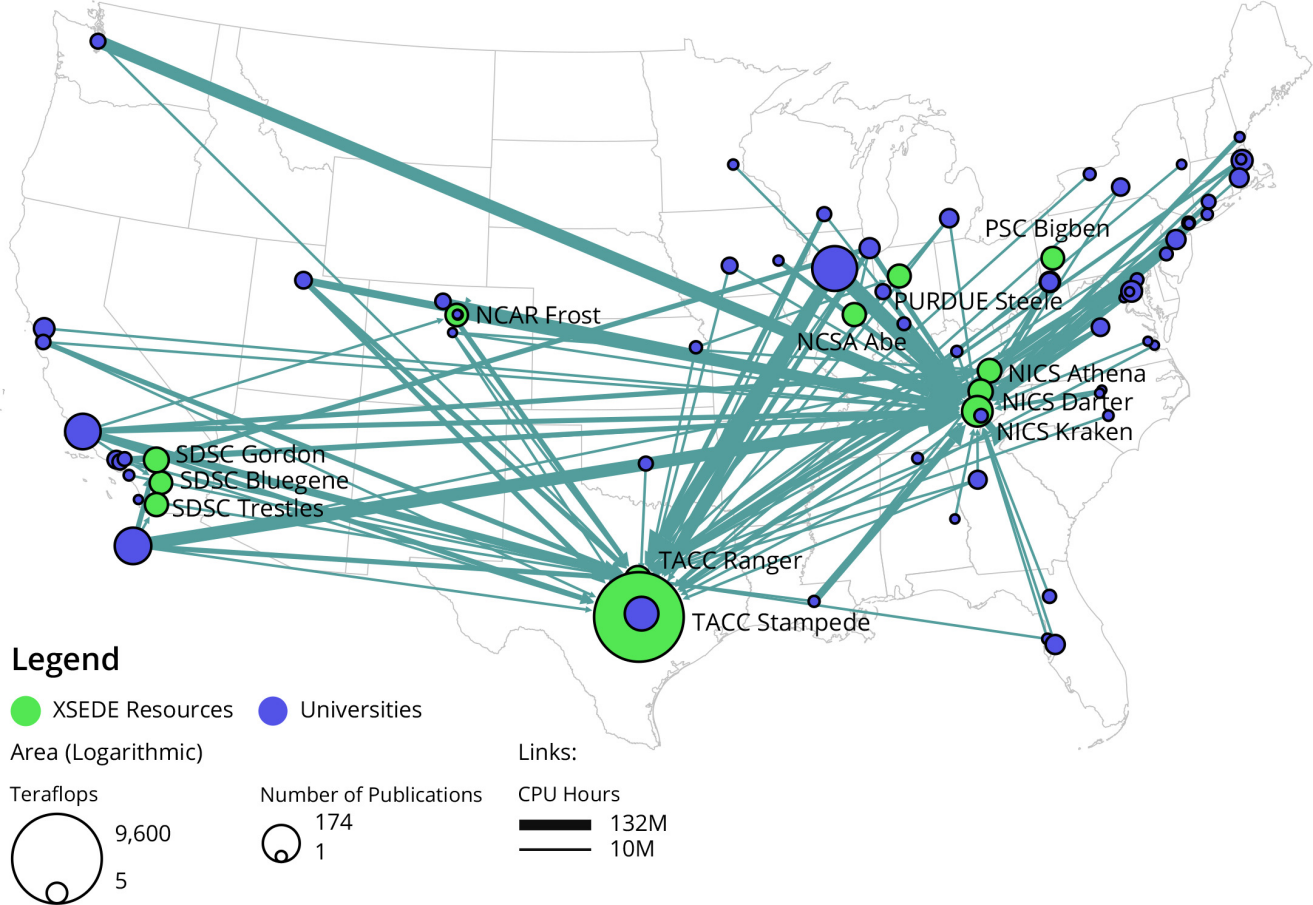
Geographic Layout of XSEDE Resources and University Consumers.

Organizational usage of XSEDE comes from a broad range of universities from many geolocations. In order to understand geographical relationships between XSEDE resources and consumers, both were overlaid on a U.S. map. In order to reduce complexity, universities using fewer than 100,000,000 CPU hours were excluded from the network, and the resulting network was plotted by location on a map. Fig 5 shows the geographic layout of XSEDE consumers (universities in blue and area size coded by number of projects) and producers (resources area size coded by number of Teraflops in thousands) with links denoting resource use (line thickness denotes number of CPU hours used in thousands). As can be seen, projects are broadly distributed geographically and the largest projects make use of resources independent of location. That is, XSEDE users do not make use of social contacts in order to improve usage or queue standing on systems that are local to them, rather they use resources with significant amounts of computing power and complete work on those resources. This is also borne out by the significant number of fields supported by the two largest resources in the bipartite network in Fig 4: Kraken and Stampede.

## 6. Discussion

The examination of XSEDE resources, consumption patterns, and publications presented in this paper helps quantify the relationship between cyberinfrastructure in support of basic research and the scientific products of that research. Data analyses and visualizations demonstrate that usage follows system capability—project computing jobs tend to go to larger resources. Plus, the usage of larger resources is dominated by users from traditional HPC fields: physics, chemistry, astronomy, and materials sciences. XSEDE is an organization under tension: the NSF promotes large-scale, big data applications on the one hand, and also promotes the support of “long tail” science and broader engagement activities that involve research fields which have not traditionally be computationally oriented. XSEDE and the Service Providers (and those proposing to become Service Providers) will need to choose carefully what kinds of usage they support.

Several XSEDE staff members were invited to comment on the analyses and visualizations and to envision their usage in daily decision making. Answers comprised the following statements:

- “This map [Fig 5] largely reflects R1 institutions, if you were to add EPSCoR (Experimental Program to Stimulate Competitive Research) institutions to this map, they would all fall in the blank spaces.”
- “The visualizations [Figs 2 and 5] show where the NSF should steer its funds” meaning, to states with lower levels of research funding.
- When discussing the Fig 5 with an XSEDE staff member who works to increase diversity of the XSEDE user base, s/he noted that high resource usage describes only one modality of use (i.e. users running jobs which consume a high portion of resources at a time, using parallel processing such as OpenMPI, or highly-parallel jobs), while there might be other types of usage—processing with R or python that generally do not require highly-parallel computation and usually have lower CPU hour requirements—that would help improve our understanding of different communities of users.

While the publication list provided by XSEDE is based on self-report, all of the reporting researchers are motivated to list their publications with XSEDE, presumably because they feel that their work on XSEDE has been successful and that the cyberinfrastructure has supported their activities effectively. Therefore, the co-authorship network can be understood as a kind of XSEDE “fanbase”. This network of motivated coauthors should be utilized for XSEDE reviews, feedback, and solicitations for future directions. This is especially true given the fact that the publication database has grown considerably over the last year and the co-authorship network is much larger today. In discussions about these findings with XSEDE staff, it was suggested that the co-authorship network could be used to provide researchers with a map of potential collaborators, showing which researchers could provide bridges to new collaborative partners. An application that provided the route from one researcher to another might provide XSEDE users novel means to identify collaborators and research partners.

While this paper increases our understanding of the relationship between resources, consumers, and publications, there is still much work to be done in this area. Unfortunately, initial data products from XSEDE were based on CPU hours and the maps here are based on CPU hours instead of normalized XSEDE Service Units. Service units are a measure of CPU usage normalized to a standard hour of usage on a common CPU model. Because of multiple resources with a broad range of processors implemented in TeraGrid and XSEDE, the actual work completed in a CPU hour can vary widely. In order to normalize usage counts across multiple systems, the standardized SU is based off of each system's performance (details on SU factors are available at https://portal.xsede.org/knowledge-base/-kb/document/bazo). Future analyses of this type should be based on normalized Service Units rather than CPU Hours. Because of the size and density of the networks generated from the XSEDE data, the presented analyses emphasize the top users of the systems. Given NSF’s interest to support “long tail” science, there should be important trends in the area of smaller projects and domains, and analyses of these projects should give more information about how resources are used by these new communities.

Further investigations in this direction can make use of other cyberinfrastructure organizations’ metrics collected from usage data as well publication information. Organizations such as the Partnership for Advanced Computing in Europe (PRACE), the Open Science Grid (OSG), and the UK-Japan RENKEI organization all provide computational resources for users to support basic research and have considerable incentives for reporting usage and scientific output. Each of these cyberinfrastructure organizations has significant differences in the mix of fields of science supported as well as the type of computational services delivered, for example the Open Science Grid provides “High Throughput Computing” as opposed to “High Performance Computing”. By conducting a meta-study across different cyberinfrastructures, it may be possible to draw further conclusions about resource usage and how it relates to the production of scientific knowledge.
